# 气管镜介入治疗肺部手术后发生的支气管胸膜瘘的回顾性分析

**DOI:** 10.3779/j.issn.1009-3419.2024.106.03

**Published:** 2024-03-20

**Authors:** Xiaosen HUO, Yuan LI, Yanyan DONG, Lingjie BIAN, Peng AN, Hang ZOU, Lei LI, Hongwu WANG

**Affiliations:** 101121 北京，北京中医药大学东直门医院呼吸病中心; Respiratory Disease Center, Dongzhimen Hospital of Beijing University of Chinese Medicine, Beijing 101121, China

**Keywords:** 肺部手术, 支气管胸膜瘘, 气管镜介入治疗, Pulmonary surgery, Bronchopleural fistula, Bronchoscopic intervention therapy

## Abstract

**背景与目的:**

气管镜介入技术作为近年来发展的新技术，具有创伤小、安全性高及可重复性的优点。本研究旨在探讨肺部恶性肿瘤和良性疾病手术后发生的支气管胸膜瘘（bronchopleural fistula, BPF）的临床特征和气管镜下介入治疗的临床疗效，为BPF的防治提供参考方法。

**方法:**

收集2020年6月至2023年9月北京中医药大学东直门医院呼吸病中心诊治的BPF患者64例，总结BPF发生部位及瘘口特点，并采用气管镜介入治疗。对瘘口较小者（≤5 mm）进行黏膜下注射聚桂醇及氰基丙烯酸异丁酯、医用生物蛋白胶或硅胶假体封堵相结合治疗；对瘘口较大者（>5 mm）分别置入不同类型的气道金属覆膜支架和心脏封堵器；观察术前、术后的卡氏体能状态（Karnofsky performance status, KPS）评分、气促评分（shortbreath scale, SS）、体温、胸腔引流液量和白细胞计数。

**结果:**

64例患者实施全肺、肺叶或肺段切除96个，发生瘘的部位65处，瘘口74个；右肺瘘口数量（63.5%）明显多于左侧（36.5%），且右下叶支气管瘘最为常见（40.5%）。术后患者的KPS评分明显升高，而SS评分、体温、胸腔引流液量和白细胞计数明显降低，与术前比较均有统计学差异（P<0.05）。电话或住院随访1-38个月，中位生存时间为21个月。完全缓解33例（51.6%），临床完全缓解7例（10.9%），部分缓解18例（28.1%），无效6例（9.4%），总有效率为90.6%。

**结论:**

肺部手术后发生的BPF临床症状较严重，常危及生命，气管镜下介入治疗BPF是一种快速有效的治疗方法。

## Abstract (en)

**Background and objective:**

As a new technique developed in recent years, bronchoscopic intervention therapy has the advantages of minimal invasion, high safety and repeatability. The aim of this study is to investigate the clinical characteristics of bronchopleural fistula (BPF) induced by surgeries for lung malignancies or benign diseases and the effect of bronchoscopic intervention therapy for BPF, so as to provide support for prevention and treatment of BPF.

**Methods:**

Data 64 patients with BPF who were treated by bronchoscopic intervention in Respiratory Disease Center of Dongzhimen Hospital, Beijing University of Chinese Medicine from June 2020 to September 2023 were collected. Patients with fistula diameter ≤5 mm were underwent submucous injection of macrogol, combined with blocking therapy with N-butyl cyanoacrylate, medical bioprotein glue or silicone prosthesis. Patients with fistula diameter >5 mm were implanted with different stents and cardiac occluders. Locations and characteristics of fistulas were summarized, meanwhile, data including Karnofsky performance status (KPS), shortbreath scale (SS), body temperature, pleural drainage volume and white blood cell count before and after operation were observed.

**Results:**

For all 64 patients, 96 anatomic lung resections including pneumonectomy, lobectomy and segmentectomy were executed and 74 fistulas occurred in 65 fistula locations. The proportion of fistula in the right lung (63.5%) was significantly higher than that in the left (36.5%). Besides, the right inferior lobar bronchial fistula was the most common (40.5%). After operation, KPS was significantly increased, while SS, body temperature, pleural drainage volume and white blood cell count were significantly decreased compared to the preoperative values (P<0.05). By telephone follow-up or readmission during 1 month to 38 months after treament, median survival time was 21 months. 33 patients (51.6%) showed complete response, 7 patients (10.9%) showed complete clinical response, 18 patients (28.1%) showed partial response, and 6 patients (9.4%) showed no response. As a whole, the total effective rate of bronchoscopic intervention for BPF was 90.6%.

**Conclusion:**

BPF induced by pulmonary surgery can lead to severe symptoms and it is usually life-threating. Bronchoscopic intervention therapy is one of the fast and effective therapeutic methods for BPF.

支气管胸膜瘘（bronchopleural fistula, BPF）是支气管与胸膜腔形成异常交通的少见并发症，患者可表现为发热、咳嗽、咳脓性痰或呼吸困难等严重症状，甚至危及生命。手术是常见原因，肺叶和全肺切除术后发生BPF概率分别为0.4%和1.9%^[1]^，病死率为18%-50%^[2]^。尽管BPF的治疗方法有保守治疗、气管镜介入治疗和外科治疗，但目前没有统一的共识。本研究旨在分析北京中医药大学东直门医院呼吸病中心诊治的64例BPF患者的临床资料，探讨其临床特征和气管镜介入治疗的临床效果，为BPF的防治提供参考。

## 1 资料与方法

### 1.1 临床资料

收集北京中医药大学东直门医院呼吸病中心2020年6月至2023年9月肺部恶性肿瘤（53例）和良性疾病（11例）术后发生的BPF患者64例。纳入标准：（1）接受过解剖性肺段、肺叶、复合肺叶/肺段或全肺切除手术；（2）临床表现：咳嗽、咳脓性痰、气促或发热等，患侧呼吸音减弱或消失，叩诊浊音和/或皮下气肿等；（3）胸部计算机断层扫描（computed tomography, CT）和支气管镜（日本奥林巴斯公司产品CV-290型）确诊。排除标准：（1）肺气肿、肺结核及肺部感染导致的周围型肺泡瘘；（2）严重精神心理疾病或重要脏器损害；（3）拒不配合的患者。64例患者中男性46例、女性18例，年龄20-75岁，平均年龄（53.1±17.6）岁。病因：原发性肺癌53例（82.8%），其中腺癌32例（50.0%）、鳞癌19例（29.7%）和小细胞肺癌2例（3.1%）；良性病因患者11例（17.2%），其中肺结核感染4例（6.3%）、炎性假瘤3例（4.6%）、支气管扩张2例（3.1%）、真菌和巨大淋巴结增生症各1例（1.6%）。原发性肺癌患者肿瘤原发灶-淋巴结-转移（tumor-node-metastasis, TNM）分期：I期32例、II期7例、III期10例和IV期4例。肺部术前/后化疗15例和术后放疗4例。伴随疾病：肺气肿及肺部感染24例（37.5%）、低蛋白血症18例（28.1%）和糖尿病11例（17.2%）。BPF分期^[3]^：早期（1-7 d）17例（26.6%）、中期（8-30 d）21例（32.8%）和晚期（>30 d）26例（40.6%）。本研究经北京中医药大学东直门医院伦理委员会审核批准（审批编号：2023DZMEC-455-02），所有患者均签署知情同意书。

### 1.2 治疗方法

积极控制肺部感染、纠正低蛋白血症和血糖等伴随疾病同时，进行介入手术治疗。全身麻醉后，高频喷射通气经硬质气管镜（杭州好克光电仪器有限公司产品HK-162H型）侧孔维持血氧饱和度，活检钳（南微医学科技股份有限公司）、氩气高频电刀（北京麦迪康维医疗设备有限公司产品CM-350Ar型）和二氧化碳冷冻探头（北京库蓝医疗设备有限公司产品K320型）等设备经气管镜活检孔进行操作。瘘口较小（≤5 mm）的患者（图1A-1D），在支气管残端四周黏膜下适量注射聚桂醇，以黏膜出现隆起、苍白为宜，可联合氰基丙烯酸异丁酯或医用生物蛋白胶进行组织黏合；如狭长残端的瘘口，可用硅胶假体相结合方法治疗；瘘口较大（>5 mm）的患者（图1E-1H），气管镜清理残端菌苔及坏死物后，在气管镜引导下置入气道金属覆膜支架（淮安市西格玛医用实业有限公司和南微医学科技股份有限公司）或心脏封堵器（北京华医圣杰科技有限公司）：大Y和L支架封堵主支气管残端；小Y支架封堵叶或段支气管残端；OKI支架封堵右中间干残端；房间隔和室间隔封堵器封堵较短的主/叶支气管残端。支架放置方法：气管镜下插入导丝至病变支气管，沿导丝将带有引导头的支架输送鞘送入，撤出引导头及导丝，将支架推送器送进鞘管内，释放支架。心脏封堵器放置方法：导丝和输送鞘先后通过瘘口，输送鞘将封堵器两个圆盘分别放置在瘘口胸腔端和气管端，其腰部放置在瘘口内，松解连接杆释放封堵器。

**图1:**
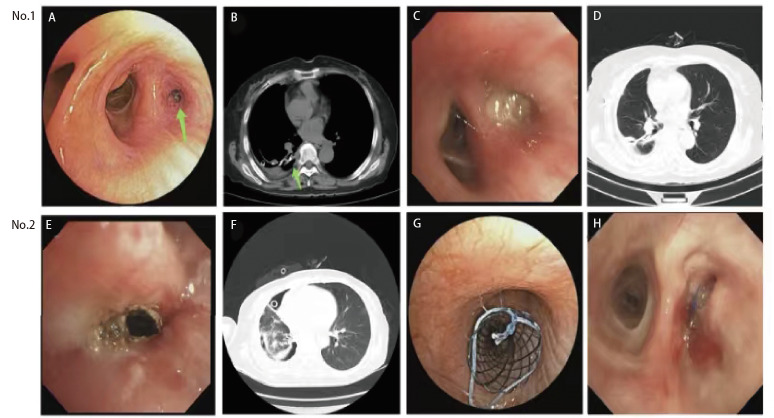
患者1，术前：A和B箭头示右下叶背段瘘口（绿色箭头）；术后：C为背段瘘口愈合；D为右余肺复张完全；患者2，术前：E为右下叶切除术后残端瘘，F为右余肺不张，并且右侧胸腔积气和积液；术后：G为右中间干放置小Y支架；H为5个月后瘘口愈合，取出小Y支架。

### 1.3 疗效评价标准^[4]^

具体评价标准见表1。有效率（%）=[完全缓解（complete response, CR）+临床完全缓解（clinical CR, cCR）+部分缓解（partial response, PR）]/[CR+cCR+PR+未缓解（no response, NR）]×100%。

**表1:** 疗效评价标准

Evaluation criteria	Fistula cured	Complete clinical remission	Stent occluded completely
CR	Yes	Yes, >1 mon	Yes
cCR	No	Yes, >1 mon	Yes
PR	No	Partial clinical remission	Stent occluded partly
NR	No	No clinical remission	No

CR: complete response; cCR: clinical CR; PR: partial response; NR: no response.

### 1.4 统计学处理

应用SPSS 21.0软件进行统计学分析，计数数据采用例数（百分比）表示，统计学分析采用χ^2^检验；计量数据采用均数±标准差或中位数表示，采用独立样本t检验或非参数检验进行分析，P<0.05表示差异有统计学意义。

## 2 结果

### 2.1 病变部位和瘘口情况

64例BPF患者中，切除全肺（左侧9个和右侧3个，12/96，12.5%）、肺叶（50/96, 52.1%）或肺段（34/96, 35.4%）共96个，发生瘘的部位65处；瘘口74个（左侧27个，36.5%；右侧47个，63.5%），且多个支气管残端部位并发多个瘘口；瘘口长径为1.0-15.0 mm，瘘口长径中位数为5.0 mm。由表2可见，右肺瘘口数量明显多于左侧，且右下叶支气管瘘占比最高（40.5%）；右下叶支气管瘘数量与左上叶比较，具有统计学差异（χ^2^=11.19, P<0.05）。根据BPF分期与瘘口长径比较，早、中和晚期瘘口长径的中位数分别为5.1、3.0和5.0 mm，组间无统计学差异（P>0.05）。通过气管镜检查，早、中期瘘口可见闭合钉或缝线脱开（7例，10.9%）、止血纱布或残端补片残留（4例，6.3%）及残端出血（1例，1.6%），而晚期瘘口可见肿瘤复发（3例，4.7%）和残端出血（1例，1.6%）。

**表2:** 肺部术后导致瘘口部位分析

Group	Cases (n)	Main bronchial fistula, n (%)	Middle bronchial fistula, n (%)	Right middle trunk fistula, n (%)	Upper lobe bronchial fistula, n (%)	Inferior bronchial fistula, n (%)
Left side	27	14 (18.9)	-	-	9 (12.2)	4 (5.4)
Right side	47	3 (4.1)	1 (1.4)	6 (8.1)	7 (9.5)	30 (40.5)

### 2.2 介入治疗情况和疗效

房间隔和室间隔封堵器封堵左主支气管和左上叶支气管各1例（2/64, 3.1%），但组织包裹紧密而未能取出封堵器；气道金属覆膜支架放置28例（28/64, 43.8%）：大Y支架10例（15.6%）和L支架1例（1.6%）封堵主支气管、小Y支架16例（25%）封堵叶或段支气管和OKI支架1例（1.6%）封堵右中间干；L支架因剧烈咳嗽咳出后被重新放置，2枚Y支架分别于置入术后14和21个月破损，并给予更换同型支架；黏膜下注射药物联合组织黏合剂及硅胶假体填塞等方法治疗34例（34/64, 53.1%）。卡氏体能状态（Karnofsky performance status, KPS）评分、气促评分（shortbreath scale, SS）、体温、胸腔引流液量和白细胞计数的术前与术后数值进行比较差异有统计学意义（P<0.05），见表3。

**表3:** 介入治疗的疗效分析

Group	KPS	SS	Body temperature (^o^C)	Pleural drainage volume (Median, mL)	White blood cell count (×10^9^/L)
Preoperation	74.61±17.40	2.03±0.79	37.58±0.68	55	10.47±3.40
Postoperation	84.77±17.44	1.11±0.80	36.57±0.31	15	6.76±1.89
P	0.001	0.001	<0.001	<0.001	<0.001

KPS: Karnofsky performance status; SS: shortbreath scale.

瘘口愈合、胸腔无残腔和胸腔引流量小于15 mL时，取出支架11例（中位留置时间为5.5个月）和拔除胸腔引流管30例（中位留置时间为2.0个月）；CR 33例（51.6%）、cCR 7例（10.9%）、PR 18例（28.1%）和NR 6例（9.4%），总有效率为90.6%。表4可见瘘口≤5 mm和>5 mm介入治疗效果；6例NR患者中肺叶切除1例、联合肺叶/段切除2例及全肺切除3例，均放置了支架；其中，因剧烈咳嗽或咯血而取出支架者3例；因肺部感染致呼吸衰竭并死亡者3例。电话或住院随访1-38个月，中位生存时间为21个月。

**表4:** 不同瘘口大小介入治疗效果

Group	Cases (n)	CR, n (%)	cCR, n (%)	PR, n (%)	NR, n (%)	Effective rate (%)
Fistula≤5 mm	34	22 (64.7)	4 (11.8)	8 (23.5)	0 (0.0)	100.0
Fistula>5 mm	30	11 (36.7)	3 (10.0)	10 (33.3)	6 (20.0)	80.0

## 3 讨论

肺部手术后发生BPF的原因是复杂多样的，按时间进行分期对分析其形成原因是有帮助的。一般而言，早期BPF与手术因素有关：闭合支气管残端组织过多或残端过长、肿瘤残留、闭合器闭合时间过短或闭合钉选择过短以及手工缝合或结扎残端不紧密等。本组病例气管镜检查未发现早期BPF肿瘤残留及残端过长现象，而存在闭合器使用方式不当、缝合或结扎不牢固及异物材料覆盖支气管残端现象。中晚期BPF的常见原因包括肿瘤浸润、辅助放疗、化疗^[5]^、肺部感染、低蛋白血症、贫血或血糖控制不佳等。补片或止血纱布覆盖支气管残端是防止BPF发生的措施，但异物的存在也是诱发BPF的因素之一；因此，术中避免无菌材料的污染是减少BPF发生的前提。我们还发现右侧肺叶术后形成瘘口数量明显多于左侧，且右下叶支气管最为常见，这与右主支气管管径粗、纵隔组织遮盖少和淋巴结易清扫等因素^[5]^有关。此外，肺叶/段切除前要充分纠正不利的伴随疾病因素，同时针对术前放化疗易导致周围组织纤维化和血流减少的因素^[6]^，应避免过度游离支气管周围组织，可利用带血管蒂的肌瓣、胸膜或心包脂肪覆盖支气管残端，能够有效地防止BPF发生^[7⇓-9]^，尤其是右下叶切除术。通过BPF分期与瘘口大小的分析，二者之间无统计学差异，说明在多种因素影响下愈合的支气管残端也会形成较大的瘘口。

BPF的治疗方法有保守治疗、气管镜介入治疗和外科治疗，目前没有统一的共识。外科治疗方式包括胸廓开窗术、胸廓成形术和使用带血管蒂的不同组织直接封堵瘘口^[7,10]^，然而外科治疗易引起较大的损伤，不仅瘘口再发率高达23.6%^[10]^，而且因呼吸衰竭和败血症导致死亡率非常高。另外，BPF发生时临床症状较严重，难以耐受立即外科手术治疗。随着气管镜介入治疗的发展，硬化剂、化学物质、封堵剂和支架或心脏封堵器等治疗方法越来越多地应用在BPF的治疗中，针对瘘口大小选择何种介入治疗方式，目前没有统一标准。有研究^[11]^报道化学物质、硬化剂和封堵剂等材料封堵<5 mm的瘘口，成功率高于≥5 mm瘘口；因此，本研究团队将5 mm作为评价瘘口大小的依据，并且已报道^[12]^35例BPF患者按照瘘口≤5 mm和>5 mm进行上述介入治疗，总有效率为88.6%。虽然在两组中不同介入方法的疗效不适合进行统计学比较，但是从数值中可发现瘘口较小者疗效更佳。今后本团队将继续扩大样本量，细化不同瘘口大小与介入治疗方法的疗效关系。不同类型的气道金属覆膜支架和心脏封堵器的选择往往根据瘘口位置、大小和残端长短等情况而定，但也有各自的特点；比如，气道金属覆膜支架覆盖了广泛的气道黏膜，阻碍了黏膜纤毛的自清能力，易引起分泌物储留；心脏封堵器虽不易引起分泌物储留和移位，但缺点在于肉芽组织包裹而不易取出封堵器。介入治疗术后KPS评分、SS评分、体温、胸腔引流液量和白细胞计数与术前比较均有显著差异，说明气管镜介入方法能够快速封堵呼吸道瘘口，减少肺部感染机会，促进余肺膨胀。胸腔引流量是评估残腔的方法之一，为避免积液潴留影响瘘口愈合，我们认为引流量小于15 mL可能是拔除引流管的指征，并且对引流管留置时间作了初步探讨。支架留置时间往往根据气管镜检瘘口是否愈合作为主要依据；值得一提的是，我们取出支架的其他原因还包括支架的破损和支架刺激肉芽组织增长而危及呼吸功能；长时间的分泌物腐蚀以及呼吸时支气管的挤压可能是导致支架破损的原因。

研究发现，本组病例中位生存时间为21个月，由此可见，气管镜下介入治疗是一种相对安全、快速和有效的方法。尽管本研究样本量有限，但目前国内类似报道尚少，研究结果仍具有一定的参考意义，我们将继续扩大样本量深入研究，以期为BPF治疗方案的选择提供更多临床依据。
